# Needles in Haystacks: Understanding the Success of Selective Pairing of Nucleic Acids

**DOI:** 10.3390/ijms23063072

**Published:** 2022-03-12

**Authors:** Carlos A. Plata, Stefano Marni, Samir Suweis, Tommaso Bellini, Elvezia Maria Paraboschi

**Affiliations:** 1 Dipartimento di Fisica ‘G. Galilei’, INFN, Università di Padova, Via Marzolo 8, 35131 Padova, Italy; cplata1@us.es (C.A.P.); samir.suweis@unipd.it (S.S.); 2 Física Teórica, Universidad de Sevilla, Apartado de Correos 1065, 41080 Sevilla, Spain; 3 Dipartimento di Biotecnologie Mediche e Medicina Traslazionale, Università di Milano, Via Fratelli Cervi 93, 20054 Segrate, Italy; stefanomarni@gmail.com (S.M.); tommaso.bellini@unimi.it (T.B.); 4 Padova Neuroscience Center, Università di Padova, Via Giuseppe Orus 2, 35131 Padova, Italy; 5 Department of Biomedical Sciences, Humanitas University, Via Rita Levi Montalcini 4, 20072 Pieve Emanuele, Italy; 6 IRCCS Humanitas Research Hospital, Via Manzoni 56, 20089 Rozzano, Italy

**Keywords:** nucleic acid interactions, pairing statistics, stat-mech modeling

## Abstract

The effectiveness of several biological and biotechnological processes relies on the remarkably selective pairing of nucleic acids in contexts of molecular complexity. Relevant examples are the on-target binding of primers in genomic PCR and the regulatory efficacy of microRNA via binding on the transcriptome. Here, we propose a statistical framework that enables us to describe and understand such selectivity by means of a model that is extremely cheap from a computational point of view. By re-parametrizing the hybridization thermodynamics on three classes of base pairing errors, we find a convenient way to obtain the free energy of pairwise interactions between nucleic acids. We thus evaluate the hybridization statistics of a given oligonucleotide within a large number of competitive sites that we assume to be random, and we compute the probability of on-target binding. We apply our strategy to PCR amplification and microRNA-based gene regulation, shedding new light on their selectivity. In particular, we show the relevance of the defectless pairing of 3${}^{\prime }$ terminals imposed by the polymerase in PCR selection. We also evaluate the selectivity afforded by the microRNA seed region, thus quantifying the extra contributions given by mechanisms beyond pairing statistics.

## 1. Introduction

The selective pairing of nucleic acids is the key molecular property enabling genetic coding, gene expression and regulation, and heredity transmission. The extent of such selectivity becomes evident in processes in which complementary strands have to selectively pair amid a plethora of other nucleic acid polymers and oligomers. Relevant examples of such a successful “needle in the haystack” search performed by nucleic acids can be found in both biological and technological contexts. For instance, in the biological context, microRNA (miRNA) play a key role in gene expression and regulation. miRNA are short RNA molecules (∼22 nt, where nt stands for nucleotides) typically targeting specific messenger RNAs (mRNA) among the molecular variety present in the cytoplasm, inducing mRNA degradation or translation halting. In the technological context, polymerase chain reaction (PCR) is the most used technique in molecular biology, allowing the exponential amplification of target DNA/cDNA regions thanks to the selective pairing between oligonucleotide primers and entire genomes/transcriptomes. In both cases, one short oligomer (of the order of 20 nt) has to search and find its complementary counterpart within much longer polymers (e.g., $∼10^{9}$ nt).

Regarding the PCR technique, since Mullis’ first publication, the primer length considered effective in PCR was in the range of 20–27 nt [1]. A simple statistical consideration is to evaluate permutations in a strand of length *L* and compare it to the total length $L_{0}$ of the analyzed genome [2]. When L=20, the possible permutation of nucleobases is around $10^{12}$, much more than the length of the human genome (around $3\times 10^{9}$). However, this simple evaluation does not take into account the possibility of forming defected pairings, which is the most relevant form of potential failure in selective targeting. In its current use, primer design is optimized through the use of algorithms that allow us to control for GC content, secondary structure, or internal complementary regions [2].

On the other hand, for miRNA selectivity, the mechanism of action has different layers of complexity. First, miRNAs in cells function within a ribonucleoprotein complex called the RNA-induced silencing complex (RISC). The formation of the mature miRNA–RISC complex is not trivial, and requires the maturation of the miRNA molecule, the association with Argonaute (AGO) proteins, and the selection of the guide strand that takes the RISC to the target mRNAs, usually in its 3${}^{\prime }$ untranslated region (3${}^{\prime }$ UTR) [3]. Moreover, although the length of mature miRNAs is ∼22 nt, the “active” region, called the “seed”, is only 6–8 nt long [4]. Generally, the seed corresponds to nucleotides 2–7, and it is considered the minimal element to bind and repress mRNA translation potential. This length must have been optimized by nature as a compromise between selectivity on the one hand and fast diffusion and accessibility to the target on the other. Despite the seed being recognized as a critical element in the miRNA mechanism, growing evidence indicates that sequences in the miRNA 3${}^{\prime }$-end play an important role in mRNA targeting [3]. Interestingly, structural studies have shown that, once the miRNA forms a complex with AGO proteins, only the seed is available to interact with the target site [5]. However, the binding of the miRNA–RISC complex to a target RNA induces a conformational change that unmasks the 3${}^{\prime }$ end of the miRNA, allowing further pairing outside the seed region [3,5], which can impact the specificity of targeting, the regulatory mechanism, and the stability of the miRNA itself. Finally, the presence of a mRNA–seed (or extended) pairing is not the only determinant of miRNA successful activity. In fact, mRNAs in cells tend to form secondary structures, and to interact with RNA binding proteins, which can limit miRNA accessibility to the target. Site accessibility was demonstrated to be a key feature for miRNA-mediated translational repression: functional miRNA target sites are preferentially located in highly accessible regions, and this feature is conserved across genomes [6]. These notions need to be taken into account in the estimate of the total amount $L_{0}$ of sites on which miRNA may bind in competition to its targets.

In spite of the differences and the complexity of the selectivity processes described above, they are both rooted in the selectivity of interactions between nucleic acids. A natural question thus arising from these remarkably successful examples of selectivity is how to model and understand these phenomena on the basis of the well-known thermodynamics of nucleic acid duplex formation [7]. Here, we tackle this problem by elaborating on a re-parametrization on three classes of base pairing error guided by the description of hybridization thermodynamics from the so-called “nearest-neighbor model” [8]. We then develop a mean field method to calculate the probability for the formation of perfect and defected duplexes in these two contexts. In particular, we focus on exploring the effect of the oligomer (primer and miRNA) length *L* in the efficiency of targeting their cognate sites within long random sequences, gaining new insights into the factors at play in both situations. The dependences on other relevant parameters such as the temperature are also analyzed.

## 2. Materials and Methods

Our strategy relies on the comparison between the Boltzmann statistical weights for on-target and off-target pairings in order to evaluate the success probability of the process. In the miRNA case, we study the pairing of the miRNA–RISC complex to the mRNA, where mainly the nucleotides within the “seed” region are available for Watson–Crick interactions; on the other hand, we consider the first annealing cycle of the PCR, being the most significant for the success of the technique.

In the following subsections, we present the main ingredients for our physical statistical description of the PCR technique and miRNA gene expression regulation: firstly, we are able to obtain the average free energy of a certain quality of duplex, thanks to a parametrization of the pairing depending on the kinds of mismatches involved. Secondly, the same parametrization allows us to obtain the degeneracy of each kind of duplex, i.e., the total number of sequences with which the primer/miRNA can realize a duplex with the same combination of mismatched bases. For our purposes of general validity of the results, we neglect the sequence specificity of the genomic ssDNA or of the mRNA and we consider them as random sequences, where the 4 nitrogenous bases are equiprobable in each nucleotide of the off-target sites. Finally, we have combined the binding free energy and the degeneracy to compute the Boltzmann weight of the on-target and off-target pairings. Comparing these two terms, we obtain the on-target pairing probability.

### 2.1. Free Energy for Duplex Formation

Differently from other works on DNA hybridization focusing on the prediction of stable pairings as a function of the temperature, i.e., the study of “melting curves” [9,10,11], we would like to characterize here the probability of on-target binding of oligonucleotides in the presence of huge numbers of random competitive sites. To do so, we have to describe the binding free energy between any given pair of interacting oligomers, as well as the degeneracy of their potential pairing.

The free energy difference ΔG between a nucleic acid duplex and its free constituent sequences can be split into an enthalpic and an entropic part,

(1) $$\begin{array}{c} \Delta G=\Delta H-T\Delta S. \end{array}$$

Nevertheless, providing an accurate description of such thermodynamic parameters characterizing the interaction between nucleic acids is not an easy task. In the highly cited review by SantaLucia and Hicks [12], detailed energetic data for several DNA motifs can be found, comprising canonical Watson–Crick pairing and a long catalog of errors, including internal mismatches, terminal mismatches, terminal dangling ends, hairpins, bulges, internal loops, and multibranched loops. The extraction of such thermodynamic parameters, however, necessarily requires the knowledge of the specific bases composing the two strings, and this is information that is not possible to access typically, or it is simply unfeasible to compute when dealing with a multitude of random possible competing pairs. Moreover, since our aim is to unveil some fundamental properties based on thermodynamic arguments with a coarse-grained modeling to explain the effectiveness of selective bindings in nucleic acids, we consider that such properties do not depend on fine details such as the specific bases composing the interacting oligomers. This hypothesis is checked for specific cases (see Appendix A, and Appendix A.5 in particular), proving the robustness and range of applicability of our description. Remarkably, our assumption of two states, i.e., on–off hybridization with no intermediate state between unbound and paired, is justified for short oligomers [13], as it is in the cases considered in this study.

For these reasons, we develop here an effective energetic model that, by considering only three classes of base pairing errors and through a “mean field” approach where all possible combinations of interacting pairs are averaged, yields a simplified but yet quantitatively fair description of DNA (or RNA) hybridization.

For the sake of concreteness, let us focus on the pairing between a generic primer (an oligomer with length *L*) and a long polymer with length $L_{0}\gg L$. Specifically, $L_{0}$ is measuring the number of ways in which the first oligomer can couple to the latter (number of sites wherein it can attach). Once *L* is defined, in our description, the duplex is fully characterized through a three-component parameter vector $\vec{\alpha }=\left(\alpha_{e1},\alpha_{e2},\alpha_{i}\right)$. This vector carries the information of the number of external mismatches, $\alpha_{e1}$ and $\alpha_{e2}$, and internal mismatches, $\alpha_{i}$. The definition of $\vec{\alpha }$ thus consists of re-parametrizing the hybridization thermodynamics on three classes of base pairing errors. To this aim, we split the total enthalpy and entropy into different contributions stemming from the different interactions involved in the duplex,

(2) $$\begin{array}{cc} \Delta H(L,\vec{\alpha })= & \Delta H_{\mathrm{perf}}\left(L\right)+\Delta H_{\mathrm{dang}}\left(\alpha_{e1},\alpha_{e2}\right)+\Delta H_{\mathrm{int}}\left(\alpha_{i}\right), \end{array}$$

(3) $$\;\Delta S(L,\vec{\alpha })=\Delta S_{\mathrm{perf}}\left(L\right)+\Delta S_{\mathrm{dang}}\left(\alpha_{e1},\alpha_{e2}\right)+\Delta S_{\mathrm{int}}\left(\alpha_{i}\right)+\Delta S_{\mathrm{salt}}\left(L,\vec{\alpha },\left[Na^{+}\right]\right).$$

Above, we have separated the contributions from the perfect match, the dangling ends, and internal mismatches. Note that entropy is additionally corrected due to salt concentration $\left[Na^{+}\right]$ [14]. In the following subsections, we account for each contribution in detail.

#### 2.1.1. Perfect Match: Initiation and Nearest-Neighbor Canonical Base Pairs

Our starting point is the contribution of an ideal matched duplex. The nearest-neighbor model has been proven to provide a very good description for the enthalpy and entropy of duplexes [12]. This model starts from initiation values $\Delta H_{0}$ and $\Delta S_{0}$, which are complemented by additive contributions coming from each couple of neighboring base pairs. Such contributions depend on the specific bases considered. Nevertheless, in our coarse-grained description, we associate a single averaged contribution $\Delta H_{n-n}$ and $\Delta S_{n-n}$ to any couple of neighboring matched base pairs (see Appendix A along with Table A1 therein for further details on the averaging). Therefore, in our framework, the enthalpy and entropy of perfectly matched duplexes depend solely on the length *L* and simply read

(4) $$\begin{array}{cc} \Delta H_{\mathrm{perf}}\left(L\right) & =\Delta H_{0}+\left(L-1\right)\Delta H_{n-n}, \end{array}$$

(5) $$\begin{array}{cc} \Delta S_{\mathrm{perf}}\left(L\right) & =\Delta S_{0}+\left(L-1\right)\Delta S_{n-n}, \end{array}$$

where we have taken into account that the number of couples of neighboring base pairs is L−1.

#### 2.1.2. Dangling Ends: External Mismatches

This contribution takes into account that the duplex may happen with a certain external mismatched base pair. Moreover, if the external base is well paired, there is a stacking contribution to the free energy, due to the base of the long polymer that is next to the pair.

**Figure 1:**
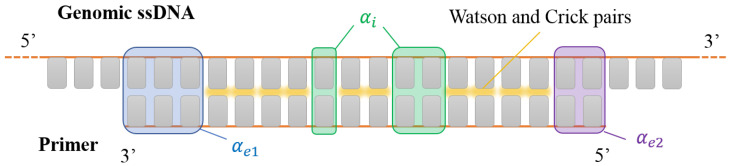
Sketch of a DNA primer interacting with a generic portion of a DNA single strand of a denaturated genome. Gray rectangles represent the nucleobases. Canonical Watson–Crick pairing is marked in yellow. Shaded boxes mark pairing defects: internal mismatches (green shades, counted by $\alpha_{i}$), terminal mismatches at the 3${}^{\prime }$ and 5’ ends (blue shades, $\alpha_{e1}$ and purple shades, $\alpha_{e2}$ respectively). In this sketch, $\alpha_{i}=3$, $\alpha_{e1}=3$ and $\alpha_{e2}=2$.

The number of external mismatches in each end is given by $\alpha_{e1}$ and $\alpha_{e2}$, respectively. Note that, in order to obtain at least one matched base pair, we need to enforce $\alpha_{e1}+\alpha_{e2}\leq L-1$ (see Figure 1). Our work hypothesis, motivated by the values typically found [12], is that external mismatches can be thought of as two dangling bases at the same end. Therefore, due to external mismatches, (*i*) $\alpha_{e1}+\alpha_{e2}$ neighboring base pairs are canceled out with respect to the perfect match and (ii) there is an extra contribution stemming from the first bases within a dangling end. Although, in reality, this contribution would depend on the identity of the bases, we consider an averaged contribution $\Delta H_{d}$ and $\Delta S_{d}$ to any dangling end (see Appendix A along with Table A2 therein for further insight on these values).

**Figure 2:**
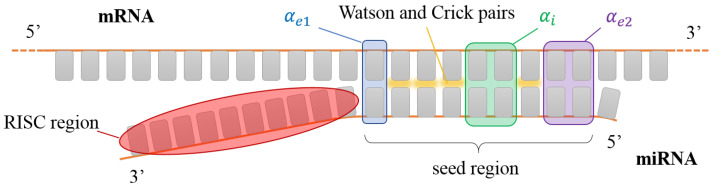
Sketch of a miRNA seed interacting with a generic portion of a mRNA. The nucleobases involved in the interaction with the AGO protein (red shading) are not available for pairing. Colored boxes have the same color code as Figure 1. In this sketch, $\alpha_{i}=2$, $\alpha_{e1}=1$ and $\alpha_{e2}=2$.

Therefore, by summing up the previous discussion, the contribution of external mismatches can be parametrized as follows:

(6) $$\begin{array}{cc} \Delta H_{\mathrm{dang}}\left(\alpha_{e1},\alpha_{e2}\right)= & c_{d}\left(\alpha_{e1},\alpha_{e2}\right)\Delta H_{d}-\left(\alpha_{e1}+\alpha_{e2}\right)\Delta H_{n-n}, \end{array}$$

(7) $$\begin{array}{cc} \Delta S_{\mathrm{dang}}\left(\alpha_{e1},\alpha_{e2}\right)= & c_{d}\left(\alpha_{e1},\alpha_{e2}\right)\Delta S_{d}-\left(\alpha_{e1}+\alpha_{e2}\right)\Delta S_{n-n}, \end{array}$$

where $c_{d}\left(\alpha_{e1},\alpha_{e2}\right)$ takes the possible values {2,3,4} depending on the external mismatches

(8) $$\begin{array}{c} c_{d}\left(\alpha_{e1},\alpha_{e2}\right)=\left\{\begin{array}{cc} 2 & \mathrm{if}\;\alpha_{e1}=\alpha_{e2}=0\\ 3 & \begin{array}{c} \mathrm{if}\;\alpha_{e1}+\alpha_{e2}>0\\ \;\;\;\mathrm{and}\;\alpha_{e1}\alpha_{e2}=0, \end{array}\\ 4 & \mathrm{if}\;\alpha_{e1}\alpha_{e2}>0, \end{array}\right. \end{array}$$

corresponding, respectively, to dangling ends without external mismatches, dangling ends plus external mismatches in one end, and dangling and external mismatches in both ends. When writing the cases above, we have kept in mind the binding of a primer inside a specific region of a longer DNA as in Figure 1. Nevertheless, this has to be modified if one is interested in studying selection by miRNA. As described in the Introduction, the active region of miRNA is finite, as represented in Figure 2. Therefore, when considering miRNA, we always assume $c_{d}=4$, regardless of the number of external mismatches.

#### 2.1.3. Internal Mismatches

Now, we consider the effect of internal mismatches in the duplex. The integer parameter $\alpha_{i}$ gives the number of internal mismatches within the duplex. When $\alpha_{i}>0$, the set of possible $\vec{\alpha }$ defining a possible duplex, with one matched base pair at least, fulfills the condition $\alpha_{e1}+\alpha_{e2}+\alpha_{i}\leq L-2$ (see Figure 1). Besides the corresponding couples of neighboring base pairs that are canceled out, the contribution penalty that stems from single internal mismatches has been thoroughly studied [12]. This depends on the particular bases. Again, following the philosophy of our coarse-grained approach, we give an averaged contribution $\Delta H_{i}$ and $\Delta S_{i}$ to those eventualities (see Appendix A along with Table A3 therein for further details on the averaging). We assume that such a contribution does not vary when more than one internal mismatch is considered. Moreover, in order to prevent further complexity, we completely neglect the internal structure of the internal mismatches (number, sizes, and separation of adjacent internal mismatches). Specifically, we consider that the effects of additional internal mismatches are equivalent to considering those mismatches to be non-consecutive. Therefore, the thermodynamic parameters associated with internal mismatches are

(9) $$\begin{array}{cc} \Delta H_{\mathrm{int}}\left(\alpha_{i}\right)= & 2\alpha_{i}\left[\Delta H_{i}-\Delta H_{n-n}\right], \end{array}$$

(10) $$\begin{array}{cc} \Delta S_{\mathrm{int}}\left(\alpha_{i}\right)= & 2\alpha_{i}\left[\Delta S_{i}-\Delta S_{n-n}\right]. \end{array}$$

According to our modeling, each internal mismatch replaces two couples of next-neighbor canonical base pairs with two next-neighbor couples of mismatched base pairs. Note that we have carried out a strong approximation in the study of internal mismatches. Nevertheless, since the states with a significant statistical weight are those with low numbers of errors, we can argue that this approximation will not lead to significant errors. The results we present in this work are reasonable and physically sound, corroborating that our assumptions do not seem to misguide our analysis.

#### 2.1.4. Salt Correction

Thermodynamic parameters are computed for a given referential salt concentration, usually 1 M of NaCl. Either excess or a defect of salt, or the presence of other ions, will imply a change in those parameters, affecting mainly the entropic contribution. Salt correction has been studied in detail in the literature [14]. In a nutshell, the most accepted proposals for this contribution assume that $\Delta S_{\mathrm{salt}}$ is a function of the salt concentration $\left[Na^{+}\right]$, usually through its logarithm. Again, this contribution has a dependence on the specific sequence that we neglect through averaging (see Appendix A for details).

#### 2.1.5. CG Contribution

In order to complement our averaged description, we develop also a more detailed, yet simple, approach that takes into account also the effect of different sequences. Specifically, we assume that the energetic parameters will be a function on the fraction of bases C or G in the DNA sequence, which may change in a significant way the thermal stability of the duplex. This description will be primarily of interest for the PCR pairing statistics, since it will highlight how the choice of specific sequences can influence the success of the PCR.

Herein, we follow the IUPAC-IUB notation, where the bases are classified as either strong bases S = {C, G} or weak bases W = {A, T}. Then, we define $f_{S}$ as the fraction of S bases in the DNA sequence of interest. Our hypothesis is that the contribution coming from the next-neighbor canonical couples of base pairs is a function of this fraction. Specifically, we consider a linear interpolation (see Appendix A for further details), i.e.,

(11) $$\begin{array}{cc} \Delta H_{n-n}\left(f_{S}\right) & =f_{S}\;\Delta H_{\left(S,S\right)}+\left(1-f_{S}\right)\;\Delta H_{\left(W,W\right)}, \end{array}$$

(12) $$\begin{array}{cc} \Delta S_{n-n}\left(f_{S}\right) & =f_{S}\;\Delta S_{\left(S,S\right)}+\left(1-f_{S}\right)\;\Delta S_{\left(W,W\right)}. \end{array}$$

When illustrating the effect of differences in the richness of strong bases, we will present the results in terms of the number of S bases $n_{CG}=Lf_{S}$.

### 2.2. Degeneracy of Equivalent Duplexes

Given a specific sequence, there is only one well-defined complementary sequence, which corresponds with $\vec{\alpha }=\vec{0}$. On the contrary, with the same specific referential sequence, we can find many duplexes with the same $\vec{\alpha }$, i.e., duplexes with errors are degenerate.

Since there are 4 different possible bases, if we focus on one of them, there is only 1 exact complementary and 3 possible mismatches. Therefore, the degeneracy of a duplex with errors made by a selective molecule (primer or miRNA) of length *L* within a specific site of a much longer nucleic acid characterized by $\vec{\alpha }$ is

(13) dL,α→=3(αe1+αe2+αi)L−2−αe1−αe2αi,

where the binomial coefficient takes into account all possible combinations of the $\alpha_{i}$ mismatches in the internal region of the duplex. Note that the simplicity of this degeneracy is partially due to our disregarding of the internal structure of internal mismatches.

### 2.3. Quantifying Selectivity

In the annealing phase of PCR, a short primer of length *L* can pair to its complementary target or to an off-target site in the two genomic ssDNA. Similarly, this also occurs in the pairing of miRNA, which can pair to its specific target or to other available sites within mRNA different molecules. The specificity of this binding is key to guarantee the success of the selective process. Herein, we compute the probability of having such successful binding using our model.

Let us consider a duplex comprising one selective molecule (primer/miRNA) and a longer nucleic acid. This duplex has, in principle, many ways to be formed. Obviously, we expect that there is a preferred binding, which corresponds with the selective molecule binding to the target region of the longer nucleic acid. For generalization purposes, let us assume that this target region appears $N_{tar}$ times in the longer nucleic acids.

The statistical weight of occurrence for a specific binding *j* is given by the Boltzmann factor

(14) $$\zeta_{j}=\exp\left(-\frac{\Delta G_{j}}{RT}\right),$$

where $\Delta G_{j}$ is the free energy difference corresponding to such binding, *R* is the gas constant, and *T* the temperature used in the experiment. Therefore, if we label j=0 as the desirable hybridization of the primer/miRNA with a specific target region, the probability of having a successful selection is

(15) $$\phi_{0}=\frac{N_{tar}\zeta_{0}}{\sum_{j}\zeta_{j}},$$

where the sum is carried out over all possible pairings in the system. Note that $\phi_{0}$ is the conditional probability of having a successful binding, given that a binding occurs. In other words, we implicitly assume that in typical conditions, concentration and temperature grant a good degree of PCR primer (or miRNA) binding to the longer nucleic acids. $\phi_{0}$ should not be confused with a melting curve, e.g., $\phi_{0}=0.1$ means that, out of a total of $n_{b}=10$ bound primers/miRNA per long polymer, $n_{b}\phi_{0}=1$ is on-target and $n_{b}\left(1-\phi_{0}\right)=9$ are off-target.

When computing $\phi_{0}$, we have conjectured that the oligomers (primer/miRNA) in the system are mutually independent, i.e., they do not compete for the binding on each specific site. Therefore, we are requiring implicitly that the total number of actual bindings $n_{b}$ per long polymer measured by the melting curve should not be much larger than $N_{tar}/\phi_{0}$. This constraint means that, on average, the number of primers/miRNA on target computed from $\phi_{0}$, i.e., $\phi_{0}\times n_{b}$, does not exceed the number of target spots on the genome. In Appendix B, we provide a numerical check of $n_{b}$ in typical genomic PCR conditions, based on the assumption of independence of primers and the computation of the melting curve, validating our hypothesis. Thus, we interpret $\phi_{0}$ as a good estimator of pairing selectivity, expressing the ratio between on-target and off-target bindings.

In order to compute $\phi_{0}$, we need to quantify the different $\zeta_{j}$. On the one hand, we can compute $\zeta_{0}$ through $\Delta G_{0}$ using the formalism introduced in the previous section considering $\vec{\alpha }=\vec{0}$. On the other hand, using a mean field approximation, we assign the averaged Boltzmann factor

(16) $$\zeta_{a}=\frac{\sum_{\vec{\alpha }}d\left(L,\vec{\alpha }\right)\exp\left[-\frac{\Delta G\left(L,\vec{\alpha },c_{\mathrm{NaCl}}\right)}{RT}\right]}{4^{L}}$$

to the rest of the possible bindings, where the sum over $\vec{\alpha }$ runs for all possible external and internal mismatches. The denominator in (16) comes from $\sum_{\vec{\alpha }}d\left(L,\vec{\alpha }\right)=4^{L}$, where the sum includes duplexes without a single complementary base pair. These duplexes can be considered within our energetic framework as impossible bindings to which we associate ΔG→∞. These off-target pairs have a weight proportional to the total sites of pairing $L_{0}$ available in the system, i.e., the number of bases of the long polymer. Finally, we can rewrite the probability in (15) for pairing to the targets that are found in number $N_{tar}$ in the system as

(17) $$\phi_{0}=\frac{N_{tar}\;\zeta_{0}}{N_{tar}\;\zeta_{0}+L_{0}\zeta_{a}}.$$

Note that we have used that $L_{0}\gg N_{tar}$, which is true for both PCR and miRNA.

This statistical approach allows us to provide a simple theoretical result with no knowledge of the specific sequences involved, which is computationally cheap. Although we are aware of the quantitative limitations of such an approach, we show here that our framework leads to a better understanding of the physics involved in selective processes such as the PCR technique or miRNA.

## 3. Results

The theoretical framework introduced above enables the evaluation of the probability of successful binding in the two conditions we have identified as especially challenging for the selectivity. In this section, we compute, for both PCR and miRNA, the targeting efficiency as a function of the relevant parameters (i.e., the length of the oligonucleotides *L*, the temperature *T*, the number of competing sites $L_{0}$, and the number of target sites in the system $N_{tar}$), by varying one parameter at a time and holding the other values fixed, and chosen to mimic typical real conditions.

The energetic parameters used in the calculations are obtained by averaging over the DNA and RNA thermodynamic dataset of the nearest-neighbor model, as detailed in Appendix A (Table A1, Table A2 and Table A3).

### 3.1. PCR

The application of our general framework to the selectivity of primers in PCR requires some specifications. First, primers are typically designed to pair to a single target position on the genome, i.e., $N_{tar}=1$. Second, in evaluating PCR efficiency, it is crucial to include the notion that the DNA polymerase needs a correct pairing between the target molecule and the 3${}^{\prime }$ terminal of the primer in order to start the amplification reaction [15]. This can be included in the model by splitting the average $\zeta_{a}$ of off-target pairings into the weighted combination of the two contributions stemming from $\alpha_{e1}=0$ and $\alpha_{e1}>0$,

(18) $$\begin{array}{cc} \;\zeta_{\left(\alpha_{e1}=0\right)} & =\frac{\sum_{\alpha_{e2}}\sum_{\alpha_{i}}d\left(L,(0,\alpha_{e2},\alpha_{i})\right)\zeta \left(0,\alpha_{e2},\alpha_{i}\right)}{4^{L-1}}, \end{array}$$

(19) $$\begin{array}{cc} \zeta_{\left(\alpha_{e1}>0\right)} & =\frac{\sum_{\alpha_{e1}>0}\sum_{\alpha_{e2}}\sum_{\alpha_{i}}d\left(L,\vec{\alpha }\right)\zeta \left(\vec{\alpha }\right)}{3\times 4^{L-1}}, \end{array}$$

where the denominator expresses the degeneracy of duplexes in the two cases. Accordingly, the total statistical weight of the pairing of the primer along the genome becomes

(20) $$\sum_{j}\zeta_{j}≃\zeta_{0}+\frac{1}{4}L_{0}\zeta_{\left(\alpha_{e1}=0\right)}+\frac{3}{4}L_{0}\zeta_{\left(\alpha_{e1}>0\right)},$$

where the coefficients 1/4 and 3/4 are the frequency with which correct and defected pairing occur in the 3${}^{\prime }$ terminal nucleobase, respectively. Thus, the probabilities of the two classes of off-target pairings, with and without correct pairing at the 3${}^{\prime }$ terminal, are

(21) $$\begin{array}{c} \phi_{\left(\alpha_{e1}=0\right)}=\frac{\frac{1}{4}L_{0}\zeta_{\left(\alpha_{e1}=0\right)}}{\zeta_{0}+\frac{1}{4}L_{0}\zeta_{\left(\alpha_{e1}=0\right)}+\frac{3}{4}L_{0}\zeta_{\left(\alpha_{e1}>0\right)}}, \end{array}$$

(22) $$\begin{array}{c} \phi_{\left(\alpha_{e1}>0\right)}=\frac{\frac{3}{4}L_{0}\zeta_{\left(\alpha_{e1}>0\right)}}{\zeta_{0}+\frac{1}{4}L_{0}\zeta_{\left(\alpha_{e1}=0\right)}+\frac{3}{4}L_{0}\zeta_{\left(\alpha_{e1}>0\right)}}. \end{array}$$

Since off-target pairing with errors at the 3${}^{\prime }$ terminal inhibits the amplification, the relevant quantity expressing the selectivity of PCR is the ratio ${\tilde{\phi }}_{0}$ of on-target pairing over all the defectless 3${}^{\prime }$ primer–genome binding,

(23) $${\tilde{\phi }}_{0}=\frac{\phi_{0}}{\phi_{0}+\phi_{\left(\alpha_{e1}=0\right)}}=\frac{\zeta_{0}}{\zeta_{0}+\frac{1}{4}L_{0}\zeta_{\left(\alpha_{e1}=0\right)}},$$

i.e., the meaningful ratio is normalized with only defectless 3${}^{\prime }$ primer–genome possible pairings, as, for the other cases, the PCR would not even start its amplification process.

**Figure 3:**
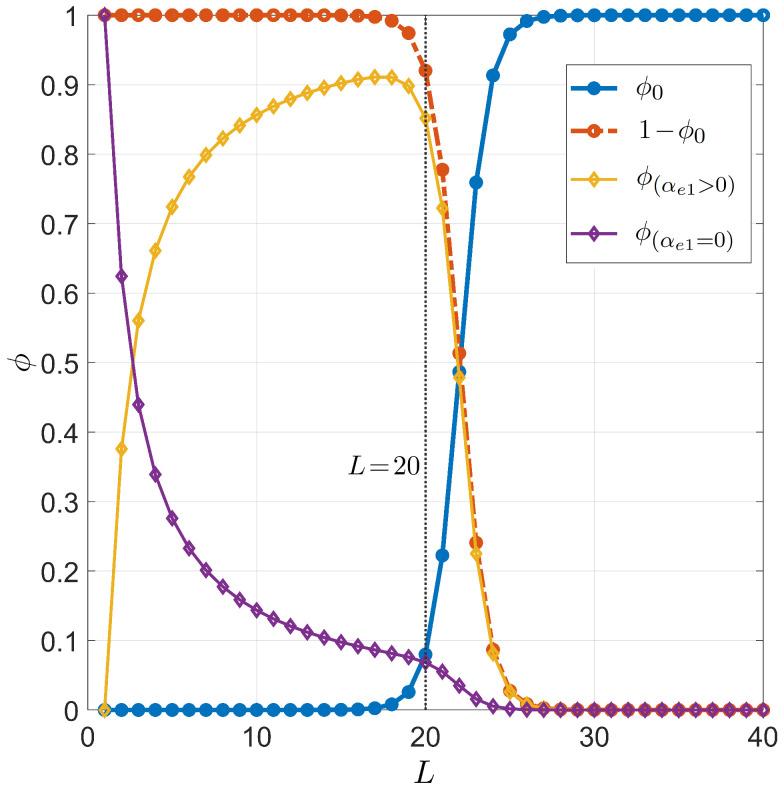
Dependence on the primer length *L* of the pairing probability for PCR. Fixed values are considered for temperature T=55 °C, total sites $L_{0}=6\times 10^{9}$, salt concentration $[Na^{+}]=55$ mM, and for CG fraction $n_{CG}=L/2$. Successful target binding ($\phi_{0}$, blue dots). Off-target binding ($1-\phi_{0}$, red dots). Off-target binding can be split into 2 contributions: off-target binding with no terminal defects at the 3${}^{\prime }$ end ($\phi_{\left(\alpha_{e1}=0\right)}$, purple diamonds), off-target binding with terminal defects at the 3${}^{\prime }$ end ($\phi_{\left(\alpha_{e1}>0\right)}$, yellow diamonds). The vertical gray line stands for L=20, a typical primer length in PCR.

**Figure 4:**
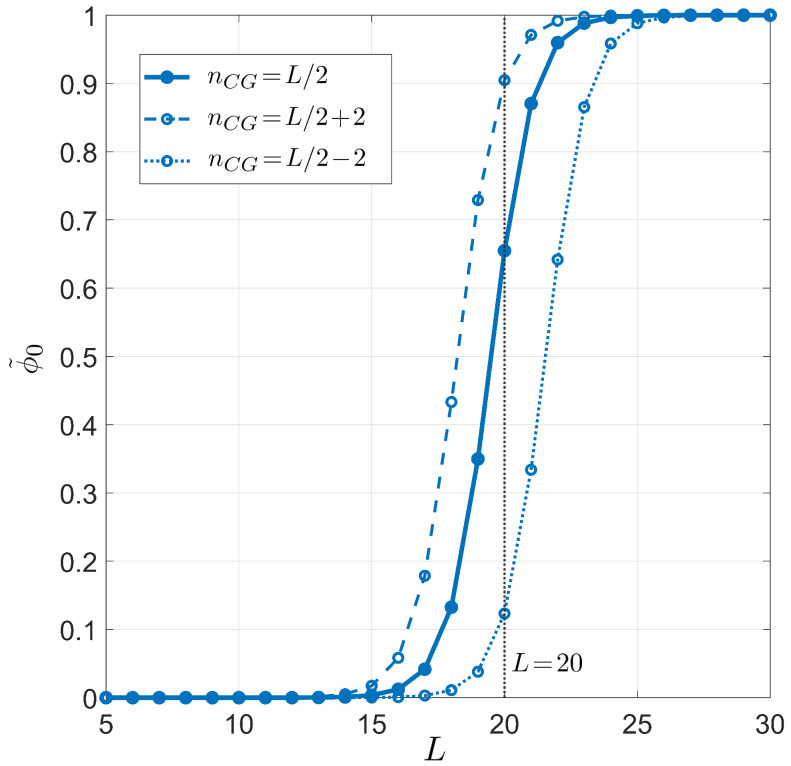
Dependence on the primer length *L* of the on-target pairing probability conditioned on the well-paired 3${}^{\prime }$ end for PCR. Fixed values are considered for temperature T=55 °C, salt concentration $[Na^{+}]=55$ mM, and total sites $L_{0}=6\times 10^{9}$, for different CG fractions in the primer. Full dots and solid line: CG fraction $n_{CG}=L/2$. Open dots and dashed line: CG fraction $n_{CG}=L/2+2$. Open dots and dotted line: CG fraction $n_{CG}=L/2-2$. Curves are computed using the average energetic description, detailed on the CG fraction (Equations (11) and (12)). The vertical gray line stands for L=20, a typical primer length in PCR.

Figure 3 and Figure 4 show the primer length dependence for the PCR pairing statistics. Specifically, we display the curves for the on-target binding probability $\phi_{0}$ (blue dots in Figure 3), the probability of off-target binding with and without 3${}^{\prime }$ pairing errors $\phi_{\left(\alpha_{e1}>0\right)}$ and $\phi_{\left(\alpha_{e1}=0\right)}$ (yellow and purple diamonds in Figure 3, respectively), and the renormalized on-target binding probability ${\tilde{\phi }}_{0}$ (Figure 4). The computation of the different curves is performed holding fixed $L_{0}=6\times 10^{9}$, since the primer can bind to both strands of the genomic DNA double helix, and T=55 °C, a typical annealing temperature; the salt concentration is $[Na^{+}]=55$ mM, representing the standard salt concentration, according to a typical DNA polymerase manufacturer’s instructions. Due to the logarithmic dependence on the salt concentration, it is necessary to notably change the salt concentration in order to observe significant changes (see Appendix C for details). Both $\phi_{0}$ and ${\tilde{\phi }}_{0}$ exhibit a rather sharp rise, indicating that the selectivity of the primer markedly changes upon lengthening or shortening the primer of a single nucleobase. The significant difference between $\phi_{0}$ and ${\tilde{\phi }}_{0}$ is due to the remarkable difference between the probability of off-target binding and its sub-ensemble of off-target with a defectless 3${}^{\prime }$ terminal (red and purple diamonds, respectively, in Figure 3). This proves that such a defect is actually quite common in random binding, since terminal defects involve the smallest energy penalties [12] with a limited growth of degeneracy. In Figure 4, we also consider the effect of modifying the fraction of CG bases in the primer. Full dots are computed with a number of CG bases $n_{CG}=L/2$; open dots correspond to $n_{CG}=L/2\pm 2$.

**Figure 5:**
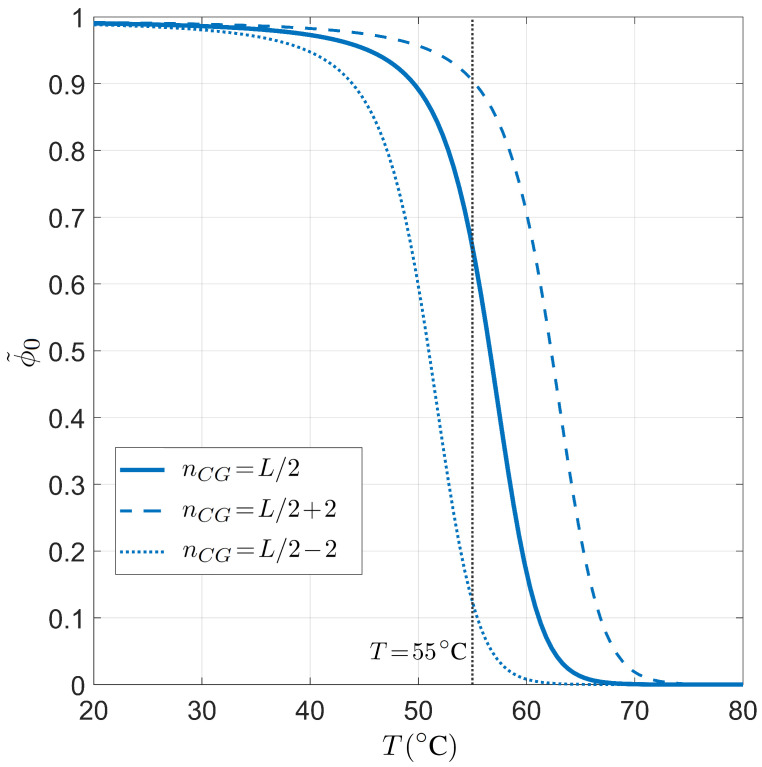
Dependence on the temperature *T* of the on-target primer pairing probability conditioned on the well-paired 3${}^{\prime }$ end for PCR. Fixed values are considered for primer length L=20, salt concentration $[Na^{+}]=55$ mM, and total sites $L_{0}=6\times 10^{9}$, for different CG fractions in the primer. Solid line: CG fraction $n_{CG}=L/2$. Dashed line: CG fraction $n_{CG}=L/2+2$. Dotted line: CG fraction $n_{CG}=L/2-2$. The gray line marks T=55 °C, a typical annealing temperature in the PCR experiments.

The temperature dependence for the PCR pairing statistics is analyzed in Figure 5. Therein, ${\tilde{\phi }}_{0}$ is plotted for either a balanced or unbalanced proportion of CG bases for L=20 and $L_{0}=6\times 10^{9}$. When *T* increases, the fraction of on-target binding decreases, as expected since the energetic gain for Watson–Crick against defected pairing decreases with *T*. Again, we find a sharp transition between high and low ${\tilde{\phi }}_{0}$, and the typical working temperature T=55 °C is indeed in the regime of high selectivity, but close to the transition to low selectivity.

**Figure 6:**
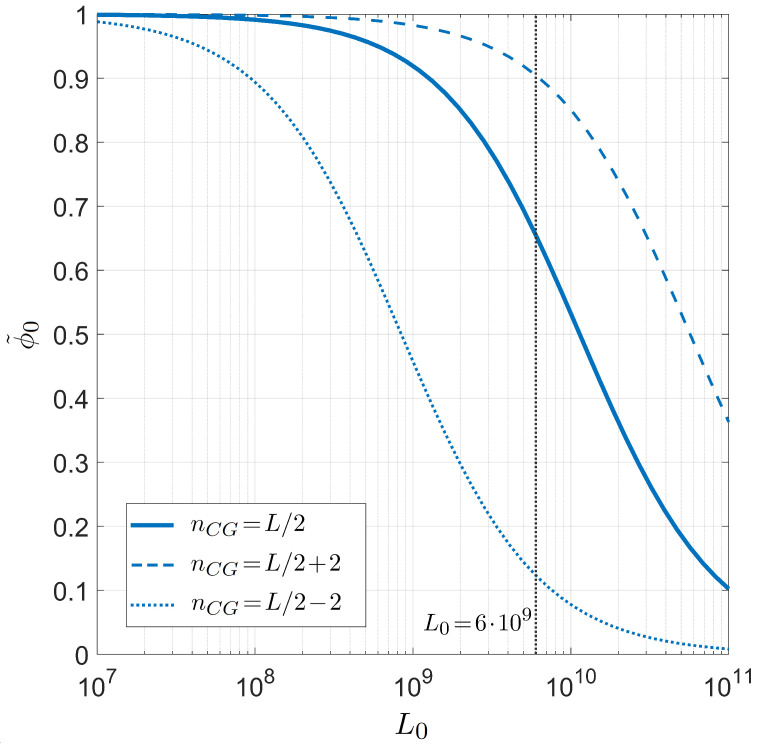
Dependence on the genome length $L_{0}$ of the on-target primer pairing probability conditioned on the well-paired 3${}^{\prime }$ for PCR. Fixed values are considered for temperature T=55 °C, salt concentration $[Na^{+}]=55$ mM, and primer length L=20, for different CG fractions in the primer. Solid line: CG fraction $n_{CG}=L/2$. Dashed line: CG fraction $n_{CG}=L/2+2$. Dotted line: CG fraction $n_{CG}=L/2-2$. The gray line marks twice the length of the human genome, $L_{0}=6\times 10^{9}$.

Finally, we compute the on-target binding probability ${\tilde{\phi }}_{0}$ as a function of the number of competing binding sites $L_{0}$. This is shown in Figure 6, where we repeat our study for different proportions of CG bases while fixing L=20 and T=55 °C. In this case, the transition is much smoother and relevant changes in the selectivity appear only when changing $L_{0}$ of order of magnitudes. When considering the $L_{0}$ of the human genome, the selectivity of the PCR primers is found to be very high, as expected.

### 3.2. miRNA

To apply our theoretical approach to the selective binding of miRNA, we need first to assess which are the most appropriate values for $L_{0}$ and $N_{tar}$.

miRNAs preferentially target 3${}^{\prime }$ UTRs, since the coding region is usually bound to other macromolecular complexes, e.g., exon junction complexes and ribosomal machinery, that would displace the RISC complex [16]. For this reason, we choose to include in our analysis only a portion of around 1000 nt, which corresponds to the median length of the 3${}^{\prime }$ UTR [17]. Moreover, to evaluate the total number of possible binding sites for each miRNA, we have to consider that not all the genes encoded in the human genome are actively transcribed within a cell. Transcriptome data in fact show that approximately 11,000 genes are simultaneously detectable within a specific cell type [18]. Thus, in evaluating the seed selectivity, we consider a reduced transcriptome length given by the product of these two quantities, $L_{0}=1.1\times 10^{7}$.

As for the evaluation of $N_{tar}$, it is relevant to notice that, differently from the PCR situation in which the primer is designed to target a single position in the genome, a single miRNA regulates the expression of several genes simultaneously. In particular, evidence suggests that the “targetome” of a miRNA is not random, but it is generally constituted by transcripts sharing the same biological network. This fact suggests that miRNAs can regulate entire target pathways [19,20]. Thus, in order to provide a reasonable value for the seed length that ensures the required selectivity, we need to consider the mean number of genes targeted by each miRNA family (groups of miRNA sharing the same seed). Analyses of preferential conservation of the seed sequence in mammals against vertebrates have indicated that the average number of targets for each miRNA family is around 300 [16]. More recent studies based on the integration of miRNA target prediction and RNA sequencing data suggest an average of 90 targets for each miRNA, highlighting the high variability among individual miRNAs [21]. Therefore, in the application of our approach to miRNA selectivity, we consider $N_{tar}$ to be in the range 100–300.

**Figure 7:**
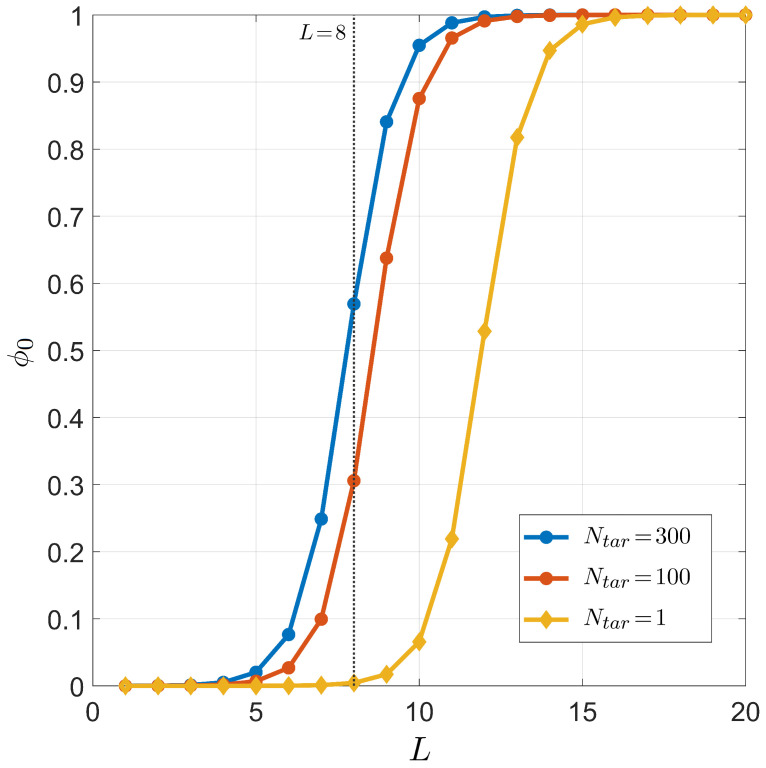
Dependence on the miRNA length *L* of the target pairing probability $\phi_{0}$ for fixed temperature T=37 °C, total sites $L_{0}=1.1\times 10^{7}$, salt concentration $[Na^{+}]=150$ mM, and CG fraction $n_{CG}=L/2$. Curves correspond to different numbers of distinct miRNA targets $N_{tar}$. Yellow dots: $N_{tar}=1$. Red dots: $N_{tar}=100$. Blue dots: $N_{tar}=300$. The gray line marks L=8, the typical length of the seed region of miRNA.

The dependence of successful binding $\phi_{0}$ on the length of the miRNA seed region is shown in Figure 7, where the three conditions of $N_{tar}=1$, $N_{tar}=100$, and $N_{tar}=300$ are considered for T=37 °C (temperature in human cell) and $L_{0}=1.1\times 10^{7}$; the salt concentration is $[Na^{+}]=150$ mM (to mimic the physiological salt concentration). The results obtained for $N_{tar}=1$ clearly indicate that, if miRNAs were meant to regulate only one specific gene, the seed length should have been 4–5 nucleobases longer in order to have the right selectivity.

It is possible to recast the effect of $L_{0}$ and $N_{tar}$ in Equation (17) in a single parameter $L_{eff}=L_{0}/N_{tar}$, which is the ratio of the number of off-target over on-target binding sites, quantifying the required selectivity. With such a definition, the equation can be rewritten as

(24) $$\phi_{0}=\frac{\zeta_{0}}{\zeta_{0}+L_{eff}\zeta_{a}}.$$

Therefore, two different systems where $N_{tar}$ and $L_{0}$ scale with the same factor, and thus with the same $L_{eff}$, are completely equivalent in our theoretical framework.

**Figure 8:**
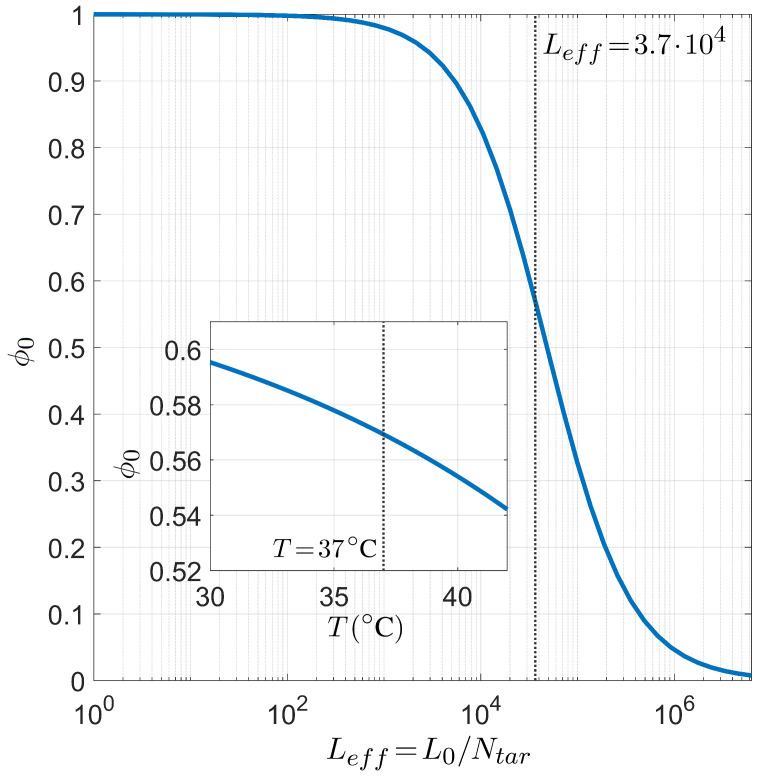
Dependence of the miRNA pairing probability $\phi_{0}$ on the reduced transcriptome length $L_{eff}$ computed with $N_{tar}=300$ for fixed temperature T=37 °C, salt concentration $[Na^{+}]=150$ mM, CG fraction $n_{CG}=L/2$, and miRNA length L=8. Inset: T dependence of $\phi_{0}$ in the same conditions and $L_{eff}=3.7\times 10^{4}$. The gray lines mark the reference values T=37 °C (inset) and $L_{eff}=3.7\times 10^{4}$ (main figure).

Finally, we present the relation between selectivity and the number of competing binding sites $L_{eff}$ in Figure 8, i.e., the analogous dependence shown in Figure 6 in the case of PCR. As already introduced above, since the role played by the length of the long polymer is always modulated by the number of targets, it suffices to study the dependence on the defined effective length $L_{eff}=L_{0}/N_{tar}$. The inset shows that the dependence on *T* of $\phi_{0}$ is not so strong as observed in the PCR case, i.e., the miRNA selectivity is less sensible to the temperature in the range of interest for the human body.

## 4. Discussion

The statistical framework developed in this work has allowed the analysis of the effectiveness of selectivity in both PCR and miRNA. In spite of its simplicity, the model has helped to better understand the relevance of the mechanisms behind the selective process, enabling non-trivial predictions that appear to have quantitative agreement with experimental observations. Among these remarkable features, we highlight the steep dependence of selectivity on *L* in Figure 3, Figure 4, and Figure 7 and the complex *L* dependence of various families of defect duplexes (Figure 3), which are discussed below separately for the two cases of interest.

### 4.1. PCR

In the context of PCR, our results convey various insights on the nature of primer selectivity. If the selective mechanism was entirely provided by on-target vs. off-target binding, i.e., expressed by $\phi_{0}$, longer primers would be needed, e.g., selectivity of $\phi_{0}>0.8$ entails L>24 from Figure 3. Nevertheless, we find that the constraint of Watson–Crick pairing at the 3${}^{\prime }$ terminal of the primers significantly changes the range of successful binding. This is because, out of the large number of expected off-target pairings (red dots in Figure 3), the fraction of primer that binds off-target with a well-formed 3${}^{\prime }$ terminal is small and has a non-trivial dependence on *L*, with a drop for L>20. When only defectless 3${}^{\prime }$ terminal binding is considered, the successful binding of L=20 primers among the plethora of off-target positions offered by the human genome is approximately ${\tilde{\phi }}_{0}≃0.65$. While this figure is still far from 1, we argue that it is sufficient, since the PCR protocol makes use of a combination of two primers, designed to target the complementary strands of the region of interest. The double strands produced at the end of the first replication cycle are much shorter than the initial genome, reducing effectively the value of $L_{0}$ in our description. Thus, in the following replication cycles, the ratio between on-target and off-target position increases, leading to a progressive increment of ${\tilde{\phi }}_{0}$.

Another outcome of our approach is the quantification of the effect of unbalancing CG and AT bases in the primer: Figure 6 shows that the reduction or addition of two CG bases markedly affects ${\tilde{\phi }}_{0}$. This is in line with the experimental procedures: when the CG content of a primer is low, its length is usually extended to compensate for the loss of selectivity.

The dependence of ${\tilde{\phi }}_{0}$ on $L_{0}$ found by our model in Figure 6 is weak, i.e., for a moderate change of $L_{0}$, the pairing probability does not change. This indicates that the PCR primer’s length granting selectivity depends weakly on the complexity of the molecular target, and thus it does not need to be significantly changed depending on the nucleic acid environment.

We have found that the on-target pairing probability decreases as *T* increases (Figure 5). This behavior is well grounded from a thermodynamic point of view, since increasing the temperature makes the free energy penalty associated with mispairing decrease, and the population of more entropic (defected) states is favored. However, this dependence appears in contradiction with the typical experience of the molecular biologist. In fact, when PCR efficiency is not very high, the annealing temperature is usually raised (typically by 2–3 °C), especially during the first cycles to improve specificity. We argue that this experimental strategy is not rooted in an increment in the selectivity at equilibrium (which is the quantity we compute), but rather it is a strategy to overcome kinetic barriers, i.e., to avoid off-target defected bindings having lifetimes comparable with the annealing time. Indeed, the increase in *T* by even a few degrees strongly reduces the lifetime of off-target bindings, thus speeding up the dynamics towards equilibrium (see Appendix D).

### 4.2. miRNA

Now, the application of our description to the miRNA selective process is discussed. The results in Figure 7 demonstrate that, in order to obtain significant selectivity over the targets around 0.8, a seed region of length 9–10 would be required, depending on the number of targets. Differently from what was found in PCR, where we obtained a primer length transition consistent with the typical experimental setting, the estimated length of the miRNA seed is larger than the actual value. This difference is not surprising since miRNAs operate within a much more intricate biological network than in vitro PCR settings.

Many factors contribute to the complexity of this system. miRNAs are part of a ribonuclear particle, where the interaction with the protein component plays an essential role not only in the mechanism of silencing that follows the binding, but also in the target recognition. Experiments exploiting AGO crosslinking and coimmunoprecipitation revealed extensive AGO-bound mRNAs in the absence of miRNA seed complementarity, thus suggesting that AGO proteins might have an RNA-binding property that allow thems to recognize mRNA targets [22]. Moreover, once the RISC complex is bound to the target mRNA, the molecular machinery undergoes a conformational change that exposes a part of the miRNA 3${}^{\prime }$ region (nts 13–16), thus allowing a supplemental pairing with the target, and providing additional selectivity [3], which could be interpreted as an increment in *L* in our description. Furthermore, the interaction between a miRNA and its target is not solely dependent on the nucleic acid pairing, but also on the availability of the target in the cell. This implies that the target gene must be transcribed, and that its secondary structure must allow the landing of the RISC complex and the binding of the miRNA to the target region. These elements suggest that the actual seed length is a compromise between the selectivity provided by nucleic acid pairing and the complexity of the cellular environment that calls for a higher degree of flexibility of the system. Overall, the comparison between the estimated and actual miRNA seed length offers a quantification of the extra selectivity brought by the mechanisms at play beyond base pairing.

## 5. Conclusions

In this study, a theoretical framework has been developed to describe selective processes in complex nucleic acid environments and applied to the PCR technique and miRNA-based gene regulation. In both cases, the selective binding occurs in spite of a huge degeneracy of competing defected pairings. The theory is constructed around two main approximations: (i) the coarse-grained description of the duplex energetics, recast based on three classes of base pairing errors, and (ii) the statistics of competing binding sites, computed by assuming random sequences.

Despite the complexity of the problem, our simple approach has led to a quite cheap model that has enabled quantitative estimates of the selectivity in the two processes, at the same time enlightening features that cannot be recognized in the absence of a quantitative framework: the sharpness of the transition in selectivity as a function of the length *L* of the oligomers at play; the relevance of the constraint of defectless 3${}^{\prime }$ terminals in PCR primer targeting; and the quantitative estimate of the contribution to miRNA target selectivity provided by processes beyond base pairing.

## Figures and Tables

**Figure A1:**
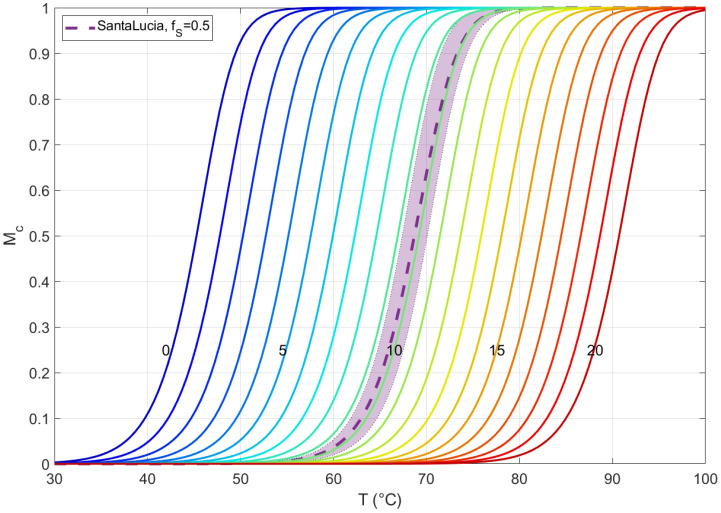
Melting curves of solutions of two 20 mers with perfect complementarity, computed with the analytical expression of a complementary system with two equipopulated species [11], with different pairing energies. The colored lines are computed with our averaged thermodynamic parameters, with number of C or G bases ranging from 0 to 20. Melting curves of 40 different duplexes with $f_{S}=0.5$ have been computed with the energetic parameters obtained with the standard NN protocol for each duplex; the average of these melting curves and the standard deviation are represented in the purple dashed line and shadow, respectively. All the solutions are in the same experimental conditions: $c_{DNA}=100$ nM and $[Na^{+}]=1$ M.

**Figure A2:**
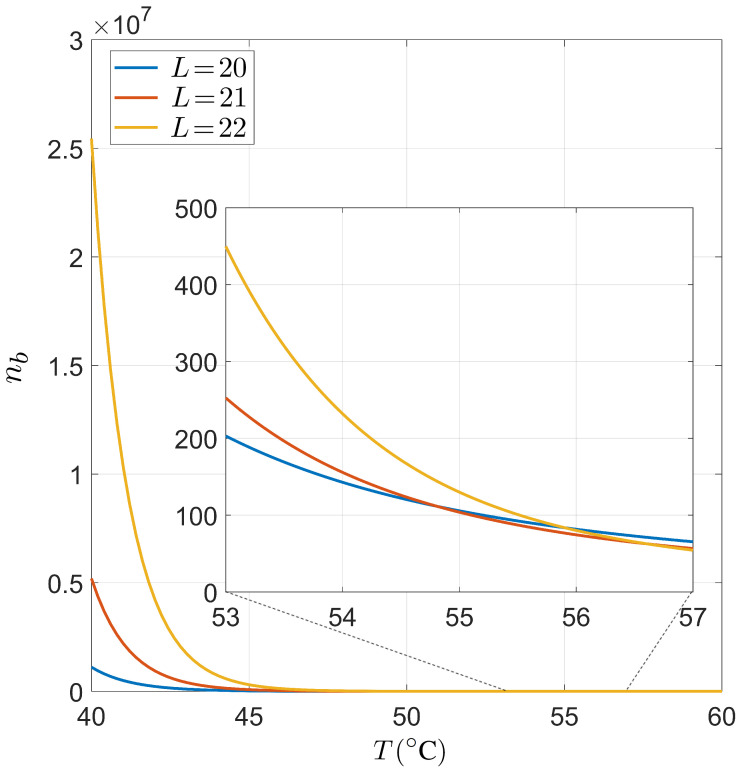
Dependence on the temperature *T* of the typical number of primers bounded per genome. We have considered fixed values for $c_{p}=400$ nM, $c_{g}=0.4$ fM, $L_{0}=6\times 10^{9}$, whereas we have considered L=20, L=21 and L=22 as typical primer lengths. The drop of $n_{b}$ around the working temperature justifies the use of the approach used in the main text.

**Figure A3:**
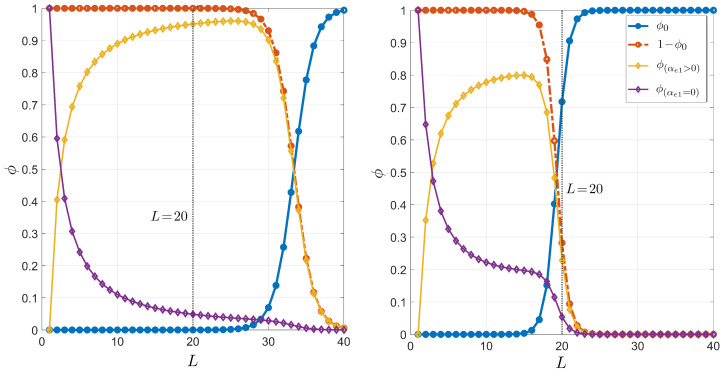
Dependence on the primer length *L* of the pairing probability for PCR. The plots are completely analogous to that shown in Figure 3 but with $[Na^{+}]=55/4$ mM (**left panel**) and $[Na^{+}]=4\times 55$ mM (**right panel**).

## Data Availability

The code used for this analysis is available upon request to the authors.

## Appendix A. Thermodynamic Parameters

In this section, we provide the values, and corresponding references from where they have been taken, which have been used in the main text for our thermodynamic description of the duplexes: either DNA/DNA in the case of the PCR or RNA/RNA in the case of miRNA. As already settled in the captions of all tables, the units we have used for ΔH and ΔS are kcal mol^−1^ and cal mol^−1^ K^−1^, respectively. If nothing is said regarding the units, we assume that everything is expressed in such units to avoid burdening the writing of the values.

### Appendix A.1. Initiation and Canonical Watson–Crick Base Pairs

Table A1 displays the thermodynamic parameters for the canonical Watson–Crick base pairing and the initiation contribution given in terms of the last bases with the end. The data for DNA/DNA interaction are taken from [12] whereas data from RNA/RNA belong to [23]. We obtain $\Delta H_{0}$, $\Delta S_{0}$, $\Delta H_{n-n}$, and $\Delta S_{n-n}$ averaging within the table. For the initiation term, we take twice the average value of the end contributions, whereas for the next-neighbor term, we have to take into account that the quadruplets that are symmetric under complementary inversion (e.g., AT/TA, TA/AT, CG/GC, and GC/CG for DNA/DNA) have half of the weight in the average since the other combinations appear twice in frequency when we consider random sequences. We obtain $\Delta H_{0}=2.4$, $\Delta S_{0}=1.2$, $\Delta H_{n-n}=-8.2$, and $\Delta S_{n-n}=-22.01$ for the PCR case (DNA/DNA interaction); and $\Delta H_{0}=7.33$, $\Delta S_{0}=9.0$, $\Delta H_{n-n}=-10.78$, and $\Delta S_{n-n}=-27.9$ for the miRNA case (RNA/RNA interaction).

### Appendix A.2. External and Internal Mismatches

In Table A2 and Table A3, we collect the thermodynamic parameters for dangling ends and internal mismatches, respectively. The assumption made in our work is that using these data for describing the RNA/RNA duplexes does not introduce significant deviations since our expectation is that the main difference between DNA/DNA and RNA/RNA comes from the different energetics of canonical Watson–Crick base pairs. The data for the dangling end have been taken from [12], whereas the information regarding internal mismatches was somewhat more split within the literature [24,25,26,27,28]. For the average description, we have simply averaged over all cells within the tables, obtaining the average contributions $\Delta H_{d}=-2.5$, $\Delta S_{d}=-6.7$, $\Delta H_{i}=0.1$, and $\Delta S_{i}=-0.8$.

### Appendix A.3. Dependence on the Sequence, Fraction of CG

Herein, we specify how we obtain the average energetic parameters for the next-neighbor couple of base pairs, considering the fraction of C or G base in the DNA sequences. We follow the IUPAC-IUB notation, where the bases are classified into two categories, either strong bases or weak ones. Specifically, we consider S = {C, G} and W = {A, T}. Then, we compute the partial average of the energetic parameters for the next-neighbor couple of base pairs, distinguishing if they are made by (W,W), (S,S), or a mixture (S,W) and (W,S). The results for such averages are displayed in Table A4. Note that $\left(\Delta H_{\left(S,S\right)}+\Delta H_{\left(W,W\right)}\right)/2=-8.25≃\Delta H_{\left(S,W),(W,S\right)}$ and $\left(\Delta S_{\left(S,S\right)}+\Delta S_{\left(W,W\right)}\right)/2=-21.9625≃\Delta S_{\left(S,W),(W,S\right)}$. These properties justify the development of a simple energetic description of the pairing based on a linear interpolation. Namely, we assign averaged next-neighbor contributions such that $\Delta H_{n-n}\left(f_{S}\right)=f_{S}\Delta H_{\left(S,S\right)}+\left(1-f_{S}\right)\Delta H_{\left(W,W\right)}$ and $\Delta S_{n-n}\left(f_{S}\right)=f_{S}\Delta S_{\left(S,S\right)}+\left(1-f_{S}\right)\Delta S_{\left(W,W\right)}$, where $f_{S}$ stands for the fraction of strong bases in the duplex of interest.

### Appendix A.4. Salt Contribution

Salt corrections, $\Delta S_{salt}$, have been adapted from Equation (22) of the article by Owczarzy et al. [14]. Therein, the effect of the salt is written for the melting temperature $T_{m}$. If we take into account that $T_{m}=\Delta H/\left(\Delta S+R\ln\left(c_{DNA}\right)\right)$, with $c_{DNA}$ being the DNA concentration, and we assume that the salt has no impact on the enthalpy, it is possible to obtain the effect of salt into the entropy. Specifically, we obtain that the salt correction to the pairing entropy depends on the salt concentration $\left[Na^{+}\right]$, on the set of parameter of the duplex presented in the article $\left(L,\vec{\alpha }\right)$, and the fraction of strong bases $f_{S}$

(A1) $$\Delta S_{salt}\left(\left[Na^{+}\right],L,\vec{\alpha }\right)=\Delta H\left(L,\vec{\alpha }\right)\left[\left(4.29f_{S}-3.95\right)\times 10^{-5}\ln\left(\left[Na^{+}\right]\right)+9.4\times 10^{-6}\times \ln^{2}\left(\left[Na^{+}\right]\right)\right],$$

being independent of the DNA concentration as expected. When the fully averaged description is used in the main text, we fix $f_{S}=0.5$.

**Table A1:** Thermodynamic parameters for canonical Watson–Crick base pairs for duplexes made by DNA/DNA (left panel) and RNA/RNA (right panel). Energy and entropy units are kcal mol^−1^ and cal mol^−1^ K^−1^, respectively.

Propagation			Propagation		
Sequence	ΔH	ΔS	Sequence	ΔH	ΔS
AA/TT	−7.6	−21.3	AA/UU	−6.82	−19.0
AT/TA	−7.2	−20.4	AU/UA	−9.38	−26.7
TA/AT	−7.2	−21.3	UA/AU	−7.69	−20.5
CA/GT	−8.5	−22.7	CA/GU	−10.44	−26.9
GT/CA	−8.4	−22.4	GU/CA	−11.4	−29.5
CT/GA	−7.8	−21.0	CU/GA	−10.48	−27.1
GA/CT	−8.2	−22.2	GA/CU	−12.44	−32.5
CG/GC	−10.6	−27.2	CG/GC	−10.64	−26.7
GC/CG	−9.8	−24.4	GC/CG	−14.88	−36.9
GG/CC	−8.0	−19.9	GG/CC	−13.39	−32.7
EC(G)/G(C)E	0.1	−2.85	EC(G)/G(C)E	1.805	−0.75
EA(T)/T(A)E	2.3	4.05	EA(U)/U(A)E	5.525	9.75

**Table A2:** Thermodynamic parameters for dangling ends for DNA/DNA interaction. Energy and entropy units are kcal mol^−1^ and cal mol^−1^ K^−1^, respectively.

		X							
**Dangling**	**Propagation**	**A**		**T**		**C**		**G**	
**End**	**Sequence**	ΔH	ΔS	ΔH	ΔS	ΔH	ΔS	ΔH	ΔS
5${}^{\prime }$-dangling	XA/T	0.2	2.3	−6.9	−20.0	0.6	3.3	−1.1	−1.5
	XT/A	−2.9	−7.7	−0.2	−0.3	−4.1	−13.2	−4.2	−15.1
	XC/G	−6.3	−17.2	−4.0	−11.0	−4.4	−12.5	−5.1	−14.1
	XG/C	−3.7	−10.1	−4.9	−13.8	−4.0	−11.8	−3.9	−10.8
3${}^{\prime }$-dangling	AX/T	−0.5	−1.2	−3.8	−12.7	4.7	14.25	−4.1	−13.2
	TX/A	−0.7	−0.7	2.9	10.3	4.4	14.8	−1.6	−3.5
	CX/G	−5.9	−16.4	−5.2	−15.1	−2.6	−7.4	−3.2	−10.3
	GX/C	−2.1	−3.8	−4.4	−13.1	−0.2	0.1	−3.9	−11.2

**Table A3:** Thermodynamic parameters for internal errors for DNA/DNA interaction. Energy and entropy units are kcal mol^−1^ and cal mol^−1^ K^−1^, respectively.

		Y							
**Propagation**		**A**		**T**		**C**		**G**	
**Sequence**	**X**	ΔH	ΔS	ΔH	ΔS	ΔH	ΔS	ΔH	ΔS
AX/TY	A	1.2	1.7	WC	WC	2.3	4.6	−0.6	−2.3
	T	WC	WC	−2.7	10.8	−1.2	6.2	1.0	0.9
	C	5.3	14.6	0.7	0.2	0.0	−4.4	WC	WC
	G	−0.7	−2.3	−2.5	−8.3	WC	WC	−3.1	−9.5
TX/AY	A	4.7	12.9	WC	WC	3.4	8.0	0.7	0.7
	T	WC	WC	0.2	−1.5	1.0	$0.7	−0.1	−1.7
	C	7.6	20.2	1.2	0.7	6.1	16.4	WC	WC
	G	3.0	7.4	−1.3	−5.3	WC	WC	1.6	3.6
CX/GY	A	−0.9	−4.2	WC	WC	1.9	3.7	−0.7	−2.3
	T	WC	WC	−5.0	−15.8	−1.5	−6.1	−4.1	−11.7
	C	0.6	−0.6	−0.8	−4.5	−1.5	−7.2	WC	WC
	G	−4.0	−13.2	−2.8	−8.0	WC	WC	−4.9	−15.3
GX/CY	A	−2.9	−9.8	WC	WC	5.2	14.2	−0.6	−1.0
	T	WC	WC	−2.2	−8.4	5.2	13.5	3.3	10.4
	C	−0.7	−3.8	2.3	5.4	3.6	8.9	WC	WC
	G	0.5	3.2	−4.4	−12.3	WC	WC	−6.0	−15.8

**Table A4:** Thermodynamic parameters for canonical Watson–Crick base pairs for duplexes made by DNA/DNA averaged over categories of bases. Energy and entropy units are kcal mol^−1^ and cal mol^−1^ K^−1^, respectively.

Propagation		
Sequence	ΔH	ΔS
WW	−7.4	−21.1
SS	−9.1	−22.8
SW(WS)	−8.2	−22.1

**Table A5:** Estimation of the relative decrease in the disassociation time $\tau_{L,\vec{\alpha }}\left(T\right)\;/\tau_{L,\vec{\alpha }}\left(T+\Delta T\right)\;$, due to a temperature increment ΔT. The values of the table are computed using Equation (A5), with different values of the increment ΔT, primer length *L*, and external mismatch $\alpha_{e1}$ in $\vec{\alpha }=\left(\alpha_{e1},0,0\right)$, representing different levels of defectiveness of the duplex. T=55 °C, as a typical PCR temperature.

		$\tau_{L,\vec{\alpha }}\left(T\right)\;/\tau_{L,\vec{\alpha }}\left(T+\Delta T\right)$					
ΔT	***L***	$\alpha_{e1}=0$	$\alpha_{e1}=1$	$\alpha_{e1}=2$	$\alpha_{e1}=5$	$\alpha_{e1}=10$	$\alpha_{e1}=15$
ΔT=+2 °C	L=20	4.4	4.2	3.8	3.1	2.1	1.4
	L=21	4.7	4.5	4.2	3.3	2.3	1.5
	L=22	5.1	4.8	4.5	3.6	2.4	1.7
ΔT=+3 °C	L=20	9.1	8.4	7.5	5.3	3.0	1.7
	L=21	10.2	9.5	8.4	6.0	3.4	1.9
	L=22	11.5	10.6	9.5	6.7	3.8	2.1

### Appendix A.5. Estimation of the Goodness of the Thermodynamic Parameters

Being aware that the pairing energy of two sequences has a significant dependence not only on the CG content but on the specific sequence of the nucleobases, herein, we analyze the error introduced in this simplification. In Figure A1, we show the melting curves of solutions of two 20 mers with perfect complementarity, all computed with the analytical expression of a complementary system with two equipopulated species [11], with different pairing energies. The colored lines are computed with our averaged thermodynamic parameters, with $f_{S}$ ranging in all the possible values. In addition, we have computed the melting curves of 40 different duplexes with $f_{S}=0.5$, with the energetic parameters obtained with the standard NN protocol for each duplex; the average of these melting curves and the standard deviation are represented in the purple dashed line and shadow, respectively. We notice that the curve with $f_{S}=0.5$ of our energetic description approximates the average melting curve with standard Santa Lucia protocol with low discrepancy ΔT<1 °C, being always within the standard deviation region, too. This is clearly a good energetic approach, because of the comparison with the standard protocol and because it provides a simple way to obtain the hybridization thermodynamics ranging across all the possible values of $f_{S}$.

## Appendix B. Melting Curve

The core of our results relies on the conditional probabilities of a specific binding $\phi_{0}$, given that there is a binding. This probability is obtained as a comparison between the Boltzmann weights $\zeta_{j}=\exp\left(-\frac{\Delta G_{j}}{RT}\right)$ for different bindings. The independence of the primers is an important hypothesis in this respect. Therein, we should guarantee that the primers do not saturate the binding locations since this could make the quantities ϕ lose their meaningfulness. Herein, we will give an estimate of the number of primers bound to any possible location per genome. To do so, we start by deriving the melting curve $M_{c}$, i.e., the fraction of free primers in the system. Resting again on the assumption of independence of the primers, which we expect to be a good approximation at least in the limit of high temperature, we take

(A2) $$M_{c}=\frac{1}{1+c_{g}\left(\zeta_{0}+L_{0}\zeta_{a}\right)},$$

with $c_{g}$ being the concentration of genomes. Note that, as seen in [11], the Boltzmann factor is accompanied by the concentration. Since, in the main text, we have always considered bound states (without comparing to the free state), the concentration was not necessary. In our approximation, the relevant concentration is the one of the genome, since we are assuming the limit of completely independent primers. Therefore, the number of expected bounded primers per genome is

(A3) $$n_{b}=\frac{c_{p}}{c_{g}}\left(1-M_{c}\right),$$

where $c_{p}$ is the concentration of primers. Our rule of thumb is that the product $\phi_{0}n_{b}$ should not be much greater than 1. Since we have obtained that $\phi_{0}≃0.1$ in the main text from Figure 3, we want to ensure that $n_{b}$ does not reach values much greater than 10.

Implementing typical values of the order of magnitude used in the experiments in Equation (A3), we obtain the results shown in Figure A2. Specifically, we have used $c_{p}=400$ nM, $c_{g}=0.4$ fM, $L_{0}=6\times 10^{9}$. Taking into account that $\phi_{0}$ was around 0.1 for L=20 and T=55 °C, and that our estimation of the bounded primers per genome is an overestimation due to the purely independence hypothesis, we can conclude that our approach is consistent with the low saturation of sites in the working temperature. Note that we obtain relatively low values for T=55 °C. This checkpoint validates the results obtained through the conditional probabilities presented in the main text.

## Appendix C. Role of the Salt Concentration

Since the dependence of the thermodynamic parameters on salt concentration is logarithmic, it is necessary to consider relatively large changes in the concentration to observe significant changes. In this appendix, we have reobtained the dependence on the primer length of the pairing probability for PCR, multiplying the typical salt concentration, 55 mM, by either a factor 1/4 or 4. The result is shown in Figure A3. As shown, the relevant crossing length between the curves corresponding to $\phi_{0}$ and $\phi_{\alpha_{e1}=0}$ decreases as the salt concentration is increased. This is a reasonable result since, generally speaking, higher salinity involves higher stability and thus significant selectivity is guaranteed even for shorter molecules.

## Appendix D. Disassociation Times

As highlighted in the main text, it is important to stress that our approach describes a system in thermodynamic equilibrium. Therein, minimum enthalpy prevails for very low temperatures, but entropic states are promoted as soon as the temperature increases. Depending on the experimental situation, it is not trivial to ensure that, for any considered situation, during the annealing stage (with a typical duration lower than 60 s), the thermodynamic equilibrium is fully reached. The disassociation time of duplexes τ, which is the inverse of the rate at which the duplexes detach, depends on the depth of the free energy barrier from the double-strand state ds to the transition state ts: $\Delta G^{‡}=\Delta H^{‡}-T\Delta S^{‡}=\Delta G_{ts}-\Delta G_{ds}$. Specifically, we assume $\tau \propto \exp\left[\Delta G^{‡}/\left(R\;T\right)\right]$.

Since the enthalpic barrier is comparable with the binding enthalpy [29], i.e., $\Delta H^{‡}≃-\Delta H$, we can estimate the temperature dependence of the typical disassociation time of a duplex as

(A4) $$\tau_{L,\vec{\alpha }}\left(T\right)\;=\tau_{0}\;\exp\left[-\Delta H\left(L,\vec{\alpha }\right)/\left(R\;T\right)\right],$$

where we have expressed the enthalpy using our parametrization of the duplex quality and $\tau_{0}$ contains the temperature-independent parameters, such as the entropic contribution to the unfolding barrier $\Delta S^{‡}$. We are interested in studying the variation in the disassociation time of a certain duplex due to a temperature change ΔT,

(A5) $$\tau_{L,\vec{\alpha }}\left(T\right)\;/\;\tau_{L,\vec{\alpha }}\left(T+\Delta T\right)\;=\exp\left[-\frac{\Delta H(L,\vec{\alpha })}{R}\left(\frac{1}{T}-\frac{1}{T+\Delta T}\right)\right],$$

where the dependence is affected exclusively by $\Delta H(L,\vec{\alpha })$. This expression enables us to compute the unfolding time variation of the duplexes, which we present in Table A5, ranging within typical conditions of the values ΔT, primer length *L*, and external mismatch $\alpha_{e1}$, representing different kinds of defectiveness of the duplex. We set T=55 °C, the same as in the main text. As we expected, the disassociation time has a significant temperature dependence for the well-paired duplexes, and a lower dependence for the duplexes with few well-paired nitrogenous bases; the main point of interest for PCR experiments is that, increasing the temperature by 2–3 °C, the typical disassociation time may be changed by an order of magnitude, with potential relevant effects on the pairing dynamics in the system.
